# Supplementary material for: Time‑resolved multi-omic analysis of paclitaxel exposure in human iPSC‑derived sensory neurons unveils mechanisms of chemotherapy‑induced peripheral neuropathy
Source: Cell Death Dis. 2026 Feb 10;17(1):211. doi: 10.1038/s41419-026-08445-2 (PMC12921266; doi:10.1038/s41419-026-08445-2)
Supplement: Supplementary file 15 — Dataset 1. [file 41419_2026_8445_MOESM15_ESM.zip › CDD_Open_Data/04_RNA_Seq/Timeline_panel_plots_etc_2023-01-23_1609.zip!/Panel_Plots_etc/14_tmod_panel_plot_msigdb_go_bp_pval_filter_0.01_pval0.05_AUC0.75_L2FC0.5_larger_letters.pdf]

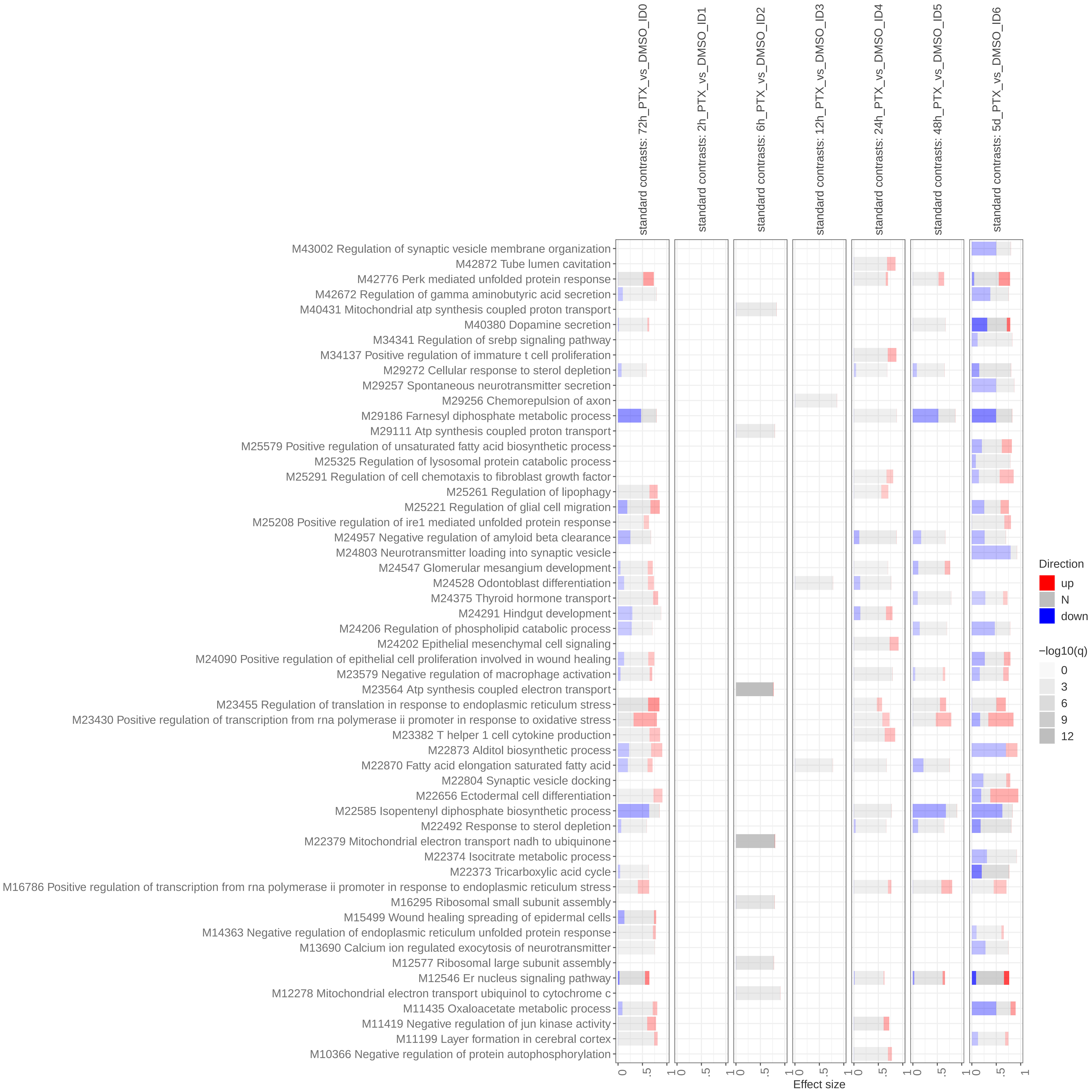

standard contrasts: 72h_PTX_vs_DMSO_ID0
standard contrasts: 2h_PTX_vs_DMSO_ID1
standard contrasts: 6h_PTX_vs_DMSO_ID2
standard contrasts: 12h_PTX_vs_DMSO_ID3
standard contrasts: 24h_PTX_vs_DMSO_ID4
standard contrasts: 48h_PTX_vs_DMSO_ID5
standard contrasts: 5d_PTX_vs_DMSO_ID6
M43002 Regulation of synaptic vesicle membrane organization
M42872 Tube lumen cavitation
M42776 Perk mediated unfolded protein response
M42672 Regulation of gamma aminobutyric acid secretion
M40431 Mitochondrial atp synthesis coupled proton transport
M40380 Dopamine secretion
M34341 Regulation of srebp signaling pathway
M34137 Positive regulation of immature t cell proliferation
M29272 Cellular response to sterol depletion
M29257 Spontaneous neurotransmitter secretion
M29256 Chemorepulsion of axon
M29186 Farnesyl diphosphate metabolic process
M29111 Atp synthesis coupled proton transport
M25579 Positive regulation of unsaturated fatty acid biosynthetic process
M25325 Regulation of lysosomal protein catabolic process
M25291 Regulation of cell chemotaxis to fibroblast growth factor
M25261 Regulation of lipophagy
M25221 Regulation of glial cell migration
M25208 Positive regulation of ire1 mediated unfolded protein response
M24957 Negative regulation of amyloid beta clearance
M24803 Neurotransmitter loading into synaptic vesicle
M24547 Glomerular mesangium development
M24528 Odontoblast differentiation
M24375 Thyroid hormone transport
M24291 Hindgut development
M24206 Regulation of phospholipid catabolic process
M24202 Epithelial mesenchymal cell signaling
M24090 Positive regulation of epithelial cell proliferation involved in wound healing
M23579 Negative regulation of macrophage activation
M23564 Atp synthesis coupled electron transport
M23455 Regulation of translation in response to endoplasmic reticulum stress
M23430 Positive regulation of transcription from rna polymerase ii promoter in response to oxidative stress
M23382 T helper 1 cell cytokine production
M22873 Alditol biosynthetic process
M22870 Fatty acid elongation saturated fatty acid
M22804 Synaptic vesicle docking
M22656 Ectodermal cell differentiation
M22585 Isopentenyl diphosphate biosynthetic process
M22492 Response to sterol depletion
M22379 Mitochondrial electron transport nadh to ubiquinone
M22374 Isocitrate metabolic process
M22373 Tricarboxylic acid cycle
M16786 Positive regulation of transcription from rna polymerase ii promoter in response to endoplasmic reticulum stress
M16295 Ribosomal small subunit assembly
M15499 Wound healing spreading of epidermal cells
M14363 Negative regulation of endoplasmic reticulum unfolded protein response
M13690 Calcium ion regulated exocytosis of neurotransmitter
M12577 Ribosomal large subunit assembly
M12546 Er nucleus signaling pathway
M12278 Mitochondrial electron transport ubiquinol to cytochrome c
M11435 Oxaloacetate metabolic process
M11419 Negative regulation of jun kinase activity
M11199 Layer formation in cerebral cortex
M10366 Negative regulation of protein autophosphorylation
0 .5 1
Effect size
Direction
up
N
down
−log10(q)
0
3
6
9
12
